# The Role of ATM in the Deficiency in Nonhomologous End-Joining near Telomeres in a Human Cancer Cell Line

**DOI:** 10.1371/journal.pgen.1003386

**Published:** 2013-03-28

**Authors:** Keiko Muraki, Limei Han, Douglas Miller, John P. Murnane

**Affiliations:** Department of Radiation Oncology, University of California San Francisco, San Francisco, California, United States of America; University of Washington, United States of America

## Abstract

Telomeres distinguish chromosome ends from double-strand breaks (DSBs) and prevent chromosome fusion. However, telomeres can also interfere with DNA repair, as shown by a deficiency in nonhomologous end joining (NHEJ) and an increase in large deletions at telomeric DSBs. The sensitivity of telomeric regions to DSBs is important in the cellular response to ionizing radiation and oncogene-induced replication stress, either by preventing cell division in normal cells, or by promoting chromosome instability in cancer cells. We have previously proposed that the telomeric protein TRF2 causes the sensitivity of telomeric regions to DSBs, either through its inhibition of ATM, or by promoting the processing of DSBs as though they are telomeres, which is independent of ATM. Our current study addresses the mechanism responsible for the deficiency in repair of DSBs near telomeres by combining assays for large deletions, NHEJ, small deletions, and gross chromosome rearrangements (GCRs) to compare the types of events resulting from DSBs at interstitial and telomeric DSBs. Our results confirm the sensitivity of telomeric regions to DSBs by demonstrating that the frequency of GCRs is greatly increased at DSBs near telomeres and that the role of ATM in DSB repair is very different at interstitial and telomeric DSBs. Unlike at interstitial DSBs, a deficiency in ATM decreases NHEJ and small deletions at telomeric DSBs, while it increases large deletions. These results strongly suggest that ATM is functional near telomeres and is involved in end protection at telomeric DSBs, but is not required for the extensive resection at telomeric DSBs. The results support our model in which the deficiency in DSB repair near telomeres is a result of ATM-independent processing of DSBs as though they are telomeres, leading to extensive resection, telomere loss, and GCRs involving alternative NHEJ.

## Author Summary

The ends of chromosomes, called telomeres, prevent chromosome ends from appearing as DNA double-strand breaks (DSBs) and prevent chromosome fusion by forming a specialized nucleo-protein complex. The critical function of telomeres in end protection has a downside, in that it interferes with the repair of DSBs that occur near telomeres. DSBs are critical DNA lesions, because if they are not repaired correctly they can result in gross chromosome rearrangements (GCRs). As a result, the deficiency in DSB repair near telomeres has now been implicated in ageing, by promoting cell senescence, and cancer, by promoting telomere dysfunction due to oncogene-induced replication stress. The studies presented here demonstrate that DSBs near telomeres commonly result in GCRs in a human tumor cell line. Moreover, our results demonstrate that the mechanism of repair of telomeric DSBs is very different from the mechanism of repair of DSBs at other locations, supporting our hypothesis that the deficiency in repair of DSBs near telomeres is a result of the abnormal processing of DSBs due to the presence of telomeric proteins. Understanding the mechanism responsible for the deficiency in DSB repair near telomeres will provide important insights into critical human disease pathways.

## Introduction

The repair of DNA double-strand breaks (DSBs) is vital for preventing gross chromosome rearrangements (GCRs) leading to cell death or cancer [1]. There are multiple mechanisms for DSB repair, including classical nonhomologous end joining (C-NHEJ) [1], homologous recombination repair (HRR) [2], and alternative nonhomologous end joining (A-NHEJ) [3]–[5]. The initial steps in DSB repair are similar for all three pathways, involving the binding of the MRE11/RAD50/NBS1 (MRN) complex to the DSB, followed by activation of ATM [6]. Phosphorylation of proteins by ATM is then instrumental in assembling a repair complex at the DSB, modifying chromatin surrounding the DSB to allow access to repair proteins, and activating cell cycle checkpoints to delay traversal through the cell cycle until repair is complete.

The primary repair mechanism for DSBs in mammalian cells is C-NHEJ, which involves the direct joining of two DNA ends, utilizing the proteins KU70, KU86, DNA-PKcs, LIG4, XRCC4, XLF, and Artemis [1]. The preference for C-NHEJ in DSB repair is insured by the ATM-mediated activation of proteins that protect of the ends of the DSB. This protection involves a variety of proteins associated with the DSB repair complex, including 53BP1 [7]–[10], histone γH2AX [11], and the MRN complex [12], [13].

When DSBs are not repaired in a timely manner, the ends of the DSB are eventually processed and resected to form single-stranded 3′ overhangs [5], [14], allowing the repair of DSBs by either HRR or A-NHEJ [2], [4]. The processing of DSBs is regulated by ATM through the activation of MRE11 [15] and CtIP [14], [16]–[18]. Following the processing of the DSB by MRE11/CtIP, resection of the 5′ end of the DSB is then mediated by EXO1 exonuclease in both yeast [19], [20] and mammalian cells [13], [21]. However, the extent of resection required, the timing in the cell cycle, and the consequences of HRR and A-NHEJ are very different. HRR requires large single-stranded 3′ overhangs to initiate repair using the complementary sequence on the sister chromatid [2], which involves activation of BRCA1 by ATM for removal of 53BP1 in late S phase and G2 [7]–[10]. Like HRR, A-NHEJ also requires the processing of DSBs by MRE11 [22]–[25] and CtIP [18], [26], [27]. MRE11 is also required for A-NHEJ in Xenopus [28] and *S. cerevisiae*, where the nuclease activity of MRE11 is necessary to release KU proteins and the MRN complex from DNA ends [12]. However, unlike HRR, DSB repair by A-NHEJ involves end joining at sites within the single stranded regions, which is often facilitated by the presence of microhomology [22], [23], [27], [29], [30], and is commonly associated with deletions [23], [31] and GCRs [26], [30]–[33]. Because of its unique characteristics, A-NHEJ is also referred to as microhomology-mediated end joining [4], [34], deletional NHEJ [24], or backup-NHEJ [5]. Although it is clear that a deficiency in C-NHEJ can promote repair of DSBs by A-NHEJ [3]–[5], it is less clear how DSBs are routed into the A-NHEJ pathway in cells that are proficient in C-NHEJ. Like HRR, ATM prevents A-NHEJ in G1 through the activation of 53BP1 and γH2AX, which work together to protect DNA ends [7]–[11]. However, unlike HRR, BRCA1 is not required for A-NHEJ, demonstrating that A-NHEJ can occur without extensive resection [18], [22]. Therefore, in addition to the inhibition of C-NHEJ, it has been pointed out that A-NHEJ can also be promoted by the stimulation of short-range MRE11/CtIP-mediated resection, the inhibition of HRR, or the inhibition of long range EXO1-dependent resection [34].

Some DSBs are more difficult to repair than others. This difference in repair efficiency is obvious from the fact that although most DSBs generated by ionizing radiation are repaired within a few hours, approximately 10 to 20% are repaired much more slowly [35]. Importantly, the DSBs that are slowly repaired are more likely to result in GCRs [36], [37]. Several factors can influence the efficiency of DSB repair. First, as originally proposed by John Ward [38], DSBs located adjacent to other radiation-induced DNA lesions, termed localized multiply damaged sites, are refractory to repair [36]. Second, DSB repair can also be influenced by chromatin structure, as shown by the fact that DSBs occurring within heterochromatin are repaired slowly and require ATM-mediated chromatin modifications that are not required for repair of DSBs that occur in euchromatin [14], [39]–[41]. Finally, as discussed below, the efficiency of DSB repair can also be influenced by the proximity of telomeres.

Telomeres are cap structures found on the ends of chromosomes that protect chromosome ends and keep them from appearing as DSBs [42], [43]. Telomeres are therefore essential for preventing chromosome fusion and genomic instability [44]–[46]. Telomeres in mammalian cells are composed of a 6 base pair repeat sequence, TTAGGG, which is added on by the enzyme telomerase [42]. TRF1 and TRF2 specifically bind to these telomeric repeat sequences and recruit RAP1, TIN2, TPP1, and POT1, which combine to generate the shelterin complex that regulates telomerase activity and protects chromosome ends [43]. Apollo exonuclease is also recruited to telomeres through the interaction with TRF2 and generates single-stranded 3′ overhangs by resection of the 5′ end of the leading strand, which is initially blunt-ended following DNA replication [47], [48]. The nuclease activity of MRE11 also contributes to the maintenance of the single-stranded 3′ overhang in a TRF2-dependent manner [49]–[51], and EXO1 nuclease functions to elongate the single-stranded 3′ overhang [52]. The single-stranded 3′ overhang is required for the association of POT1 and its partner TPP1, which promote the formation of the t-loop that is necessary for telomere end protection. The extent of processing of the end of the chromosome is limited by the binding of POT1, so that deficiencies in POT1 or TPP1 result in long single-stranded 3′ overhangs on the end of the chromosome [53]–[55].

We have previously demonstrated that telomeric regions are deficient in NHEJ [56]. A similar deficiency in NHEJ at interstitial sites containing telomeric repeat sequences led us to propose that the telomere-specific binding protein TRF2 actively suppresses C-NHEJ as part of its role in protecting the end of the chromosome [44], [56]. One model for the inhibition of NHEJ near telomeres involves the inhibition of ATM by TRF2 [57]. As at interstitial DSBs, ATM may be involved in the protection of DSBs by activation of 53BP1 and H2AX [7]–[11]. Alternatively, ATM could be required for repair of DSBs near telomeres, because subtelomeric regions are heterochromatin [58], [59], and ATM is required for DSB repair in heterochromatin [14], [39], [41]. However, we found no difference in HRR near telomeres [56], which in view of the requirement for ATM in HRR [2], suggests that ATM is functional near telomeres. We therefore proposed a second model in which the sensitivity of telomeric regions to DSBs is due to the inappropriate processing of DSBs, which would generate large single-stranded 3′ overhangs that are poor substrates for C-NHEJ [11], [51], [60], [61], but good substrates for A-NHEJ [18], [22]–[27]. In this model, DSBs within subtelomeric regions are processed to generate a single-stranded 3′ overhang in the same fashion as telomeres, which occurs through an ATM-independent process involving the regulation of the Apollo and/or MRE11 nucleases by TRF2.

Two recent studies have reported that persistent DSBs near telomeres in normal human cells in culture and *in vivo* contribute to ageing and ionizing radiation-induced senescence [62], [63]. Importantly, one of these studies showed that the ectopic localization of TRF2 caused a delay in repair of interstitial DSBs in mammalian cells, and the presence of telomeric repeat sequences inhibited NHEJ and the recruitment of the NHEJ protein LIG4 in yeast [63]. Cell senescence caused by oncogene expression was also shown recently to result from telomere dysfunction in normal human fibroblasts in culture and in preneoplastic cells *in vivo* [64]. The fact that the dysfunctional telomeres in most of the senescent cells still contained telomeric repeat sequences led the authors to conclude that irreparable DSBs near telomeres rather than telomere loss were responsible. Importantly, senescence due to telomere dysfunction was not observed in malignant tumors, consistent with our model that telomere loss resulting from oncogene-induced replication stress in tumor cells that lack of cell cycle checkpoints serves as a mechanism for GCRs in human cancer [46].

In the current study, we have investigated the effect of ATM deficiency on the consequences of DSBs near telomeres to determine how the mechanism of repair of DSBs differs at telomeric and interstitial sites. Importantly, investigating the role of ATM allowed us to determine whether the sensitivity of telomeric regions to DSBs is due to the inhibition of ATM, which could result in the loss of end protection and failure to repair DSBs, or whether it is due to the inappropriate processing of DSBs as though they are telomeres, which would be independent of ATM. Both of these mechanisms would involve the known functions of TRF2 mentioned above, and are consistent with the ability of TRF2 to inhibit DSB repair [63]. The approach we used involved generating DSBs at specific telomeric or interstitial locations with I-*Sce*I endonuclease. We employed three different assay systems used in our earlier studies, one that uses the activation of the gene for green fluorescent protein (GFP) to monitor the frequency of NHEJ, one that uses the inactivation of the GFP gene to monitor large deletions, and a PCR-based assay to monitor the frequency of small deletions [56]. In addition, we included a fourth assay system that monitors the frequency of GCRs using the activation of the DsRed gene. While this assay does not detect GCRs involving large deletions, it does provide a method for determining the relative frequency of GCRs at different locations. Using these four assay systems, we compared how the inhibition of ATM kinase activity by KU55933 or the knockdown of ATM expression by shRNA affects the types of events resulting from DSBs generated at telomeric and interstitial sites. Importantly, comparing the relative proportion of the four different types of events rules out the possibility that the results can be explained solely by a difference in the frequency of DSBs generated by I-*Sce*I at interstitial and telomeric sites. The results demonstrate that ATM is functional near telomeres and is required for the protection of DSBs, despite the fact that ATM can be inhibited by TRF2. The results also show that the large deletions resulting from DSBs near telomeres are independent of ATM, and therefore do not occur through the mechanism involved in the processing and resection of DSBs at interstitial sites. The results are therefore consistent with our model in which the sensitivity of telomeres to DSBs is due to the inappropriate processing of DSBs as though they are telomeres, which leads to extensive resection and GCRs involving A-NHEJ.

## Results

### Assays for NHEJ, GCRs, large deletions, and small deletions

**Figure 1:**
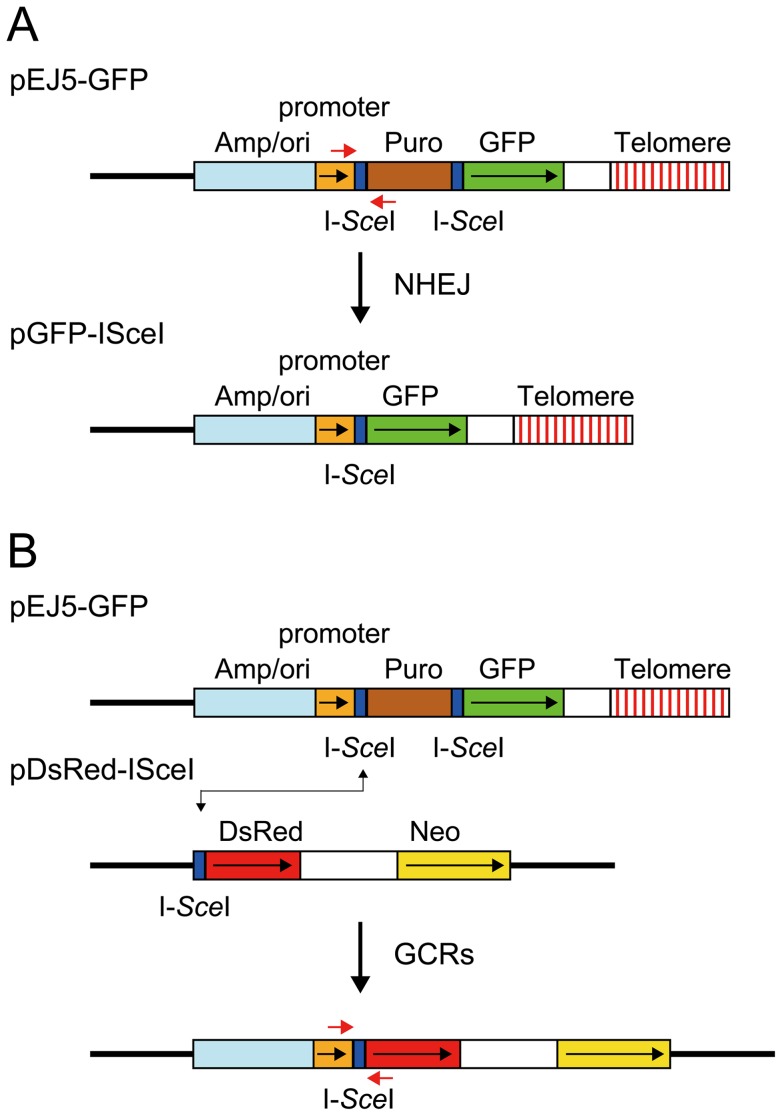
The structure of the plasmids used to monitor NHEJ, GCRs, and large deletions. (A) NHEJ was monitored using the pEJ5-GFP plasmid integrated at interstitial (not shown) or telomeric sites (shown). pEJ5-GFP contains a GFP gene that is initially inactive due to the presence of a puromycin-resistance (puro) gene located between the GFP gene and its promoter. NHEJ occurring between the distal ends of the I-*Sce*I-induced DSBs at either end of the puro gene results in the activation of the GFP gene. A PCR product generated with oligonucleotide primers spanning one of the I-*Sce*I sites in the pEJ5-GFP plasmid (red arrows) was digested with I-*Sce*I endonuclease to determine the frequency of small deletions at a single I-*Sce*I-induced DSB. Cell clones containing the pGFP-ISceI plasmid integrated at interstitial sites (not shown) or telomeric sites (shown) were used for analysis of large deletions that inactivate the GFP gene. (B) GCRs were monitored using cell clones that contain the pEJ5-GFP plasmid integrated at an interstitial (not shown) or telomeric (shown) site and the pDsRed-ISceI plasmid integrated at an interstitial site at a different location in the genome. The DsRed gene in the pDsRed-ISceI plasmid is initially inactive due to the lack of a promoter, but becomes activated following NHEJ between the I-*Sce*I-induced DSBs in the pEJ5-GFP and pDsRed-ISceI plasmids. The location of oligonucleotide primers used for PCR to analyze the junctions between the pEJ5-GFP and pDsRed-ISceI plasmids are shown (red arrows). The location of the ampicillin gene and plasmid origin of replication (Amp/ori), chicken β-actin promoter (promoter), puro gene (Puro), GFP coding sequence (GFP), and telomere are shown. Also show is the genomic DNA (solid line), directions of transcription (black arrows), and I-*Sce*I recognition sites (I-*Sce*I).

The studies presented here rely on four assays specifically designed to compare the types of events occurring as a result of DSBs at interstitial and telomeric sites. The DSBs at specific locations in these assay systems are generated with the I-*Sce*I endonuclease, which introduces DSBs at an 18 bp recognition sequence found in integrated plasmid DNA. The first assay system used in our studies determines the frequency of large deletions by monitoring the loss of GFP expression following the expression of I-*Sce*I endonuclease in cell clones containing the pGFP-ISceI plasmid (Figure 1A). The I-*Sce*I site in the pGFP-ISceI plasmid is located between the GFP coding sequence and its chicken β-actin promoter. The loss of expression of the GFP gene in cell clones containing the pGFP-ISceI plasmid requires deletions larger than 20 bps at the I-*Sce*I site, which is necessary to delete the start codon for the GFP gene or truncate the chicken β-actin promoter. This assay system is therefore capable of distinguishing larger deletions from small deletions of a few bps, which are the most frequent events at interstitial I-*Sce*I-induced DSBs [65]–[67]. We previously used this assay system to demonstrate a high frequency of large deletions at DSBs near telomeres [56]. Although not apparent from this GFP-based assay system, our previous studies involving Southern blot analysis of genomic DNA from individual subclones demonstrated that the I-*Sce*I-induced deletions near telomeres are much larger than those observed at interstitial sites, typically resulting in the loss of the entire 7 kb plasmid and the telomere [68]. In addition to large deletions, GCRs or direct telomere addition occurring at or near the I-*Sce*I site (chromosome healing) would also result in loss of GFP expression in this assay. However, the frequency of these events is very low [68]–[71]. The loss of GFP expression can also occur through changes in chromatin structure, which is increased near telomeres due to telomere position effect [59], [72], [73]. However, this does not affect the results of this assay, because although the expression of the telomeric GFP gene is gradually reduced during passage in culture, cells with complete silencing of the GFP gene are rare [56].

The second assay system used in our studies determines the frequency of NHEJ by monitoring the appearance of GFP^+^ cells following the expression of I-*Sce*I endonuclease in cell clones containing the pEJ5-GFP plasmid (Figure 1A). The activation of the GFP gene in the pEJ5-GFP plasmid results from NHEJ between the distal ends of two different I-*Sce*I sites located at either end of the puro gene, which is inserted between the GFP gene and its promoter [27]. The I-*Sce*I site may or may not be retained in the process, depending on whether the ends are directly rejoined or joined after the loss or addition of nucleotides at the DSB. We previously used this assay system to demonstrate a deficiency in NHEJ near telomeres [56].

The third assay system used in our studies determines the frequency of GCRs using the same clones containing the pEJ5-GFP plasmid that were used for the analysis of NHEJ. However, this assay system monitors the frequency of activation of the DsRed gene as a result of rearrangements in which one of the I-*Sce*I sites in the pEJ5-GFP plasmid is joined with the I-*Sce*I site in a pDsRed-ISceI plasmid that is integrated at a different location in the genome (Figure 1B). The DsRed gene in the integrated pDsRed-ISceI plasmid is initially inactive due to the absence of a transcriptional promoter, but is activated when the I-*Sce*I-induced DSB at the 3′ end of the chicken β-actin promoter in the pEJ5-GFP plasmid is joined with the I-*Sce*I-induced DSB at the 5′ end of the DsRed gene. The cell clones containing both the pEJ5-GFP and pDsRed-ISceI plasmids can therefore be used to simultaneously monitor the frequency of NHEJ (joining two I-*Sce*I sites in close proximity - green cells) and GCRs (joining two I-*Sce*I sites on different chromosomes - red cells) as a result of interstitial or telomeric I-*Sce*I-induced DSBs. A similar assay system involving the activation of a selectable *neo* gene by I-*Sce*I-induced DSBs has previously been used to investigate the mechanisms involved in the formation of chromosome translocations [26], [33], [74], [75].

The fourth assay system used in our studies determines the frequency of small deletions occurring during rejoining of the ends of one of the I-*Sce*I sites located in the pEJ5-GFP plasmid. For this assay, genomic DNA from cells expressing I-*Sce*I endonuclease is first amplified by PCR using primers that span one of the I-*Sce*I sites (Figure 1A), and the PCR product is then digested with I-*Sce*I endonuclease to determine the fraction of the PCR product that has lost the I-*Sce*I site, i.e. is not cut. The percentage of cells in the population that contain small deletions is then determined after correcting for the frequency of NHEJ and large deletions (see Materials and Methods). Using this assay system, we previously reported that there is little difference in the frequency of small deletions at interstitial and telomeric I-*Sce*I-induced DSBs [68].

### The frequency of NHEJ and GCRs at interstitial and telomeric DSBs

**Figure 2:**
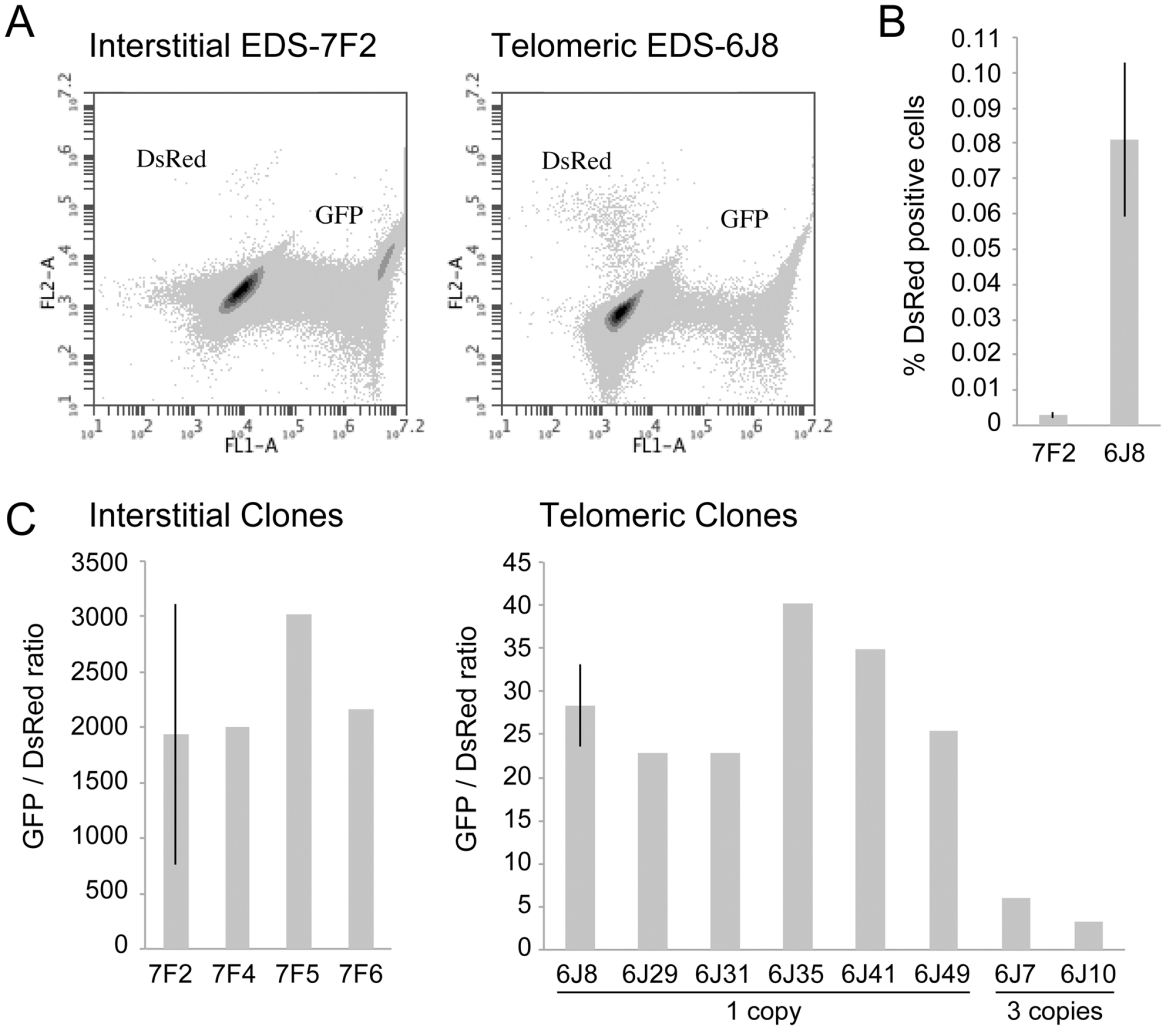
The frequency of NHEJ and GCRs at interstitial and telomeric I-*Sce*I-induced DSBs. (A) FACs analysis was used to determine the frequency of GFP^+^ (NHEJ) and DsRed^+^ (GCRs) cells in clone EDS-7F2 (with interstitial pEJ5-GFP and interstitial pDsRed-ISceI) and EDS-6J8 (with telomeric pEJ5-GFP and interstitial pDsRed-ISceI). (B) The frequency of DsRed^+^ cells was determined following infection with the pQCXIH-ISceI retrovirus and selection with hygromycin for 14 days. (C) The ratio of GFP^+^ (NHEJ) and DsRed^+^ (GCRs) cells was determined in clones that contain a single copy of the pEJ5-GFP plasmid integrated at an interstitial (EDS-7F2, EDS-7F4, EDS-7F5, EDS-7F6) or telomeric (EDS-6J8, EDS-6J29 EDS-6J31, EDS-6J35, EDS-6J46, EDS-6J49) site, and a single copy of the pDsRed-ISceI plasmid integrated at an interstitial site. Clones EDS-6J7 and EDS-6J10 contain a single copy of the pEJ5-GFP plasmid integrated at a telomeric site and 3 tandem copies of the pDsRed-ISceI plasmid integrated at an interstitial site. All samples were analyzed in triplicate. Error bars represent standard deviation of three separate experiments.

The cell clones described above that contain the pDsRed-ISceI plasmid integrated at an interstitial site and the pEJ5-GFP plasmid integrated at either an interstitial (EDS-7F) or telomeric (EDS-6J) site were used to determine the frequency of NHEJ and GCRs at interstitial and telomeric DSBs. Following infection with the pQCXIH-ISceI retrovirus and selection with hygromycin for 14 days, the percentage of cells expressing GFP or DsRed was determined by flow cytometry (Figure 2A). Consistent with our earlier studies [56], the frequency of NHEJ (GFP^+^ cells) was lower in clone EDS-6J8 with a telomeric pEJ5-GFP plasmid than in clone EDS-7F2 with an interstitial pEJ5-GFP plasmid (data not shown). In contrast, the frequency of GCRs (DsRed^+^ cells) was much greater in clone EDS-6J8 than in clone EDS-7F2 (Figure 2B). This difference in NHEJ and GCRs at interstitial and telomeric DSBs is evident from the much lower ratio of GFP^+^ to DsRed^+^ cells in EDS-6J clones containing the telomeric pEJ5-GFP gene compared to EDS-7F clones containing the pEJ5-GFP gene at an interstitial site (Figure 2C). The large standard deviation observed in the GFP^+^ to DsRed^+^ ratio in clone EDS-7F2 is a result of the extremely low level of DsRed^+^ cells in clones with an interstitial pEJ5-GFP plasmid. This low frequency of GCRs in the EDS-7F clones is consistent with the low frequency of translocations (3–5×10^−5^) previously reported to result from rearrangements between two I-*Sce*I-induced DSBs on different chromosomes [26], [33], [74], [75]. Importantly, the frequency of GCRs at telomeric DSBs in the EDS-6J clones is underestimated in our system, because it does not detect GCRs that occur in combination with large deletions, which as we have previously shown, represent the majority of rearrangements at telomeric DSBs [68], [71]. In addition, the efficient repair of the DSB in the interstitial pDsRed-ISceI plasmid will limit the frequency of GCRs detected by this assay. Proof that repair of DSBs within the interstitial pDsRed-ISceI plasmid are rate limiting is demonstrated by the fact that the EDS-6J7 and EDS-6J10 clones that contain three tandem copies of the pDsRed-ISceI plasmid have approximately a 3-fold higher frequency of DsRed^+^ cells then the EDS-6J8 clone containing a single copy of the pDsRed-ISceI plasmid (Figure 2C). Regardless of these limitations, this assay provides an analysis of the relative differences in the frequency of GCRs at different locations, and clearly shows that the deficiency in NHEJ near telomeres is associated with an increase in GCRs.

### The characterization of recombination junctions at GCRs in DsRed^+^ cells

**Figure 3:**
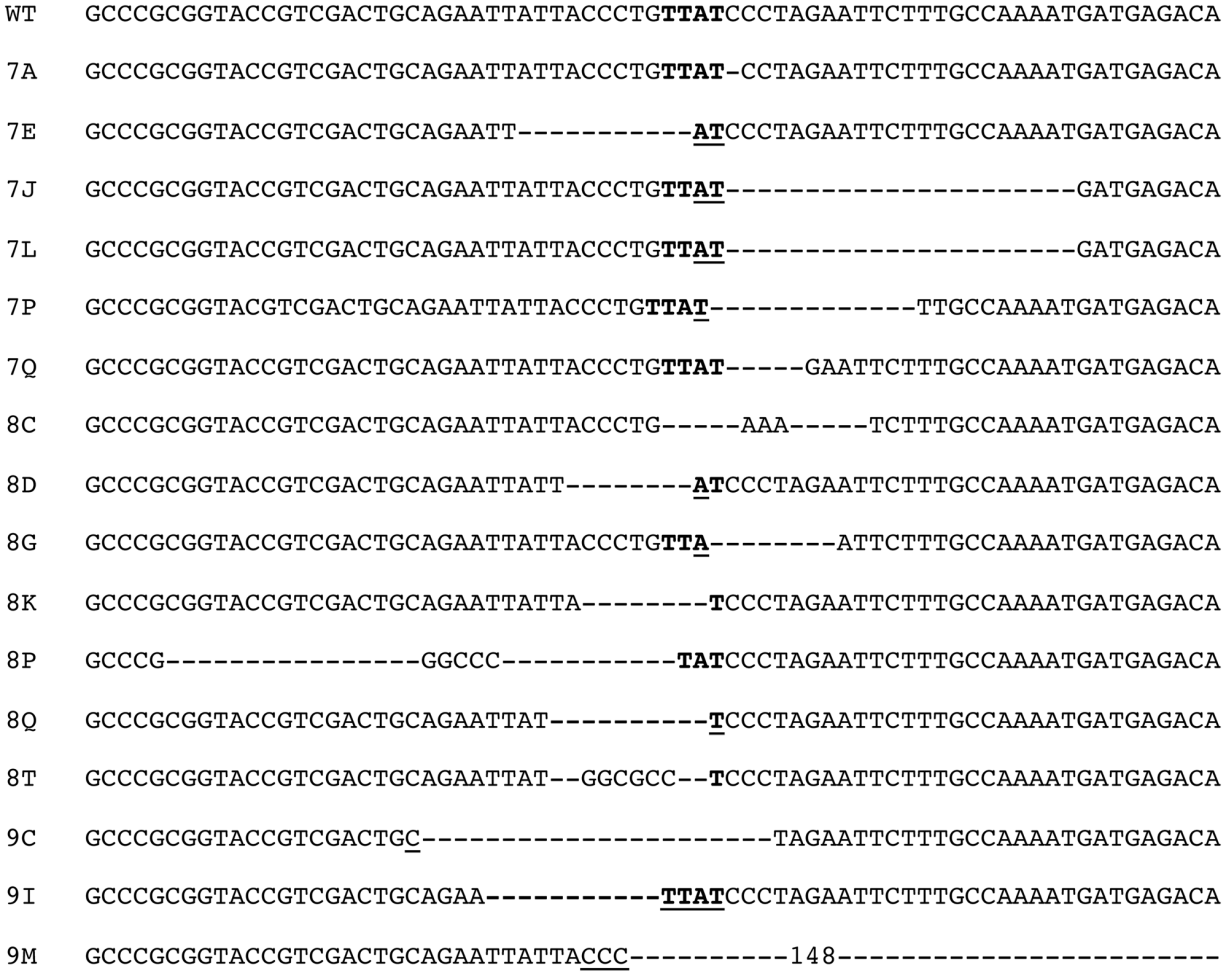
DNA sequence analysis of recombination junctions involved in the formation of GCRs in DsRed^+^ subclones. Genomic DNA was isolated from individual DsRed^+^ subclones following expression of I-*Sce*I in clones EDS-6J7 and EDS-6J8 (with telomeric pEJ5-GFP and interstitial pDsRed-ISceI). The sites containing the recombination junctions were amplified by PCR using one primer in the pEJ5-GFP plasmid and one primer in the pDsRed-ISceI plasmid (see Figure 1). DNA sequence analysis was then performed on the PCR fragments to determine the structure of the recombination junction involved in the formation of the GCR. The location of the 4-nucleotide overhang generated by I-*Sce*I endonuclease (bold), deletions at the site of the DSB (dashes), and insertions (nucleotides between dashed lines) are shown.

To confirm that the expression of the DsRed gene results from recombination between the I-*Sce*I sites in the pEJ5-GFP and pDsRed-ISceI plasmids, PCR was performed using one primer specific for the chicken β-actin promoter in the pEJ5-GFP plasmid, and one primer specific for the DsRed gene in the pDsRed-ISceI plasmid (see Figure 1B). As expected, genomic DNA from the parental EDS-6J7 and EDS-6J8 cell clones produced no PCR product (data not shown). However, following infection with the pQCXIH-ISceI retroviral vector and selection with hygromycin, both clones showed a PCR product of the size expected for NHEJ between the I-*Sce*I sites in the two plasmids (data not shown). To analyze individual recombination junctions in DsRed*^+^* cells, we performed flow sorting to isolate pooled populations of DsRed^+^ cells from clones EDS-6J7 and EDS-6J8 expressing I-*Sce*I endonuclease. The pooled populations of DsRed^+^ cells were then plated out at low density and individual colonies were selected to isolate individual subclones expressing the DsRed gene. DNA sequence analysis of the PCR products demonstrated deletions of 1 to 148 bps (average 21 bps) at the I-*Sce*I site in 16 of 17 (94%) DsRed^+^ subclones (Figure 3). Insertions of 3 to 6 bps (3 of 17 subclones, 19%) and microhomology of 1 to 4 bps (10 of 17 subclones, 59%) were also observed. The high frequency of deletions and microhomology found at the recombination junctions suggests that A-NHEJ is commonly involved in the formation of GCRs in our assay, consistent with previous studies in which A-NHEJ was found to be involved in translocations involving two different I-*Sce*I sites [26], [33], [74], [75]. Importantly, the frequency of loss of the I-*Sce*I site during the formation of GCRs in our system is greater than was previously observed during NHEJ at interstitial DSBs (40%), but is similar to the frequency of loss of the I-*Sce*I site during NHEJ at telomeric DSBs (60%) [56], suggesting that A-NHEJ is involved in DSB repair near telomeres.

### The effect of ATM deficiency on large deletions at interstitial and telomeric DSBs

**Figure 4:**
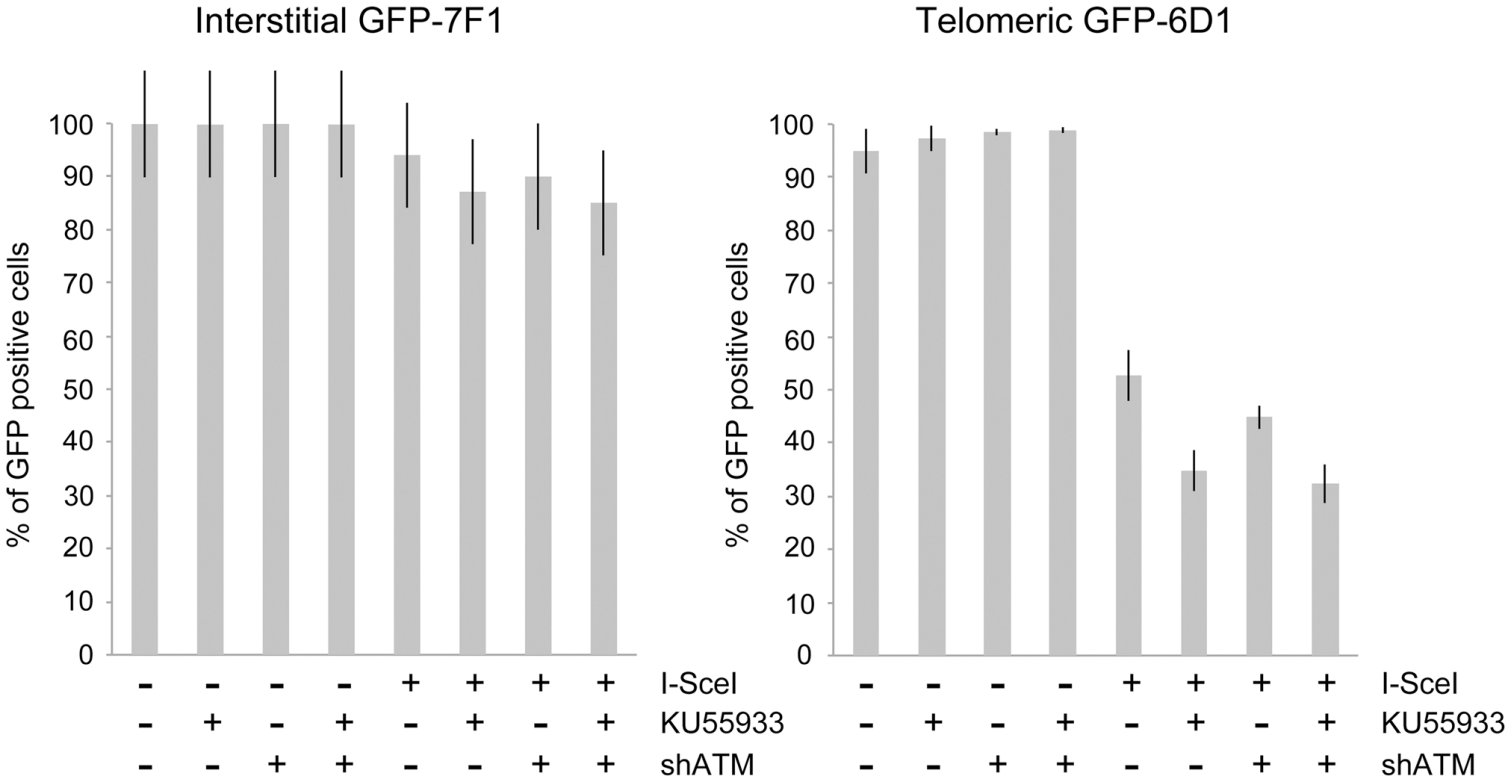
The effect of ATM deficiency on large deletions at interstitial and telomeric DSBs. The frequency of GFP-negative cells (large deletions) was determined in clones GFP-7F1 (with interstitial pGFP-ISceI) and GFP-6D1 (with telomeric pGFP-ISceI) following the infection with either the pQCXIH control retrovirus vector or the pQCXIH-ISceI retrovirus vector and selection in hygromycin for 14 days. All samples were analyzed in triplicate. Error bars represent standard deviation of three separate experiments.

Consistent with our earlier studies [56], [68], the expression of I-*Sce*I endonuclease in clone GFP-7F1 resulted in large deletions (loss of GFP expression) at interstitial DSBs in 6.0% of the cells, while I-*Sce*I-induced DSBs near a telomere in clone GFP-6D1 resulted in large deletions in 47.3% of the cells (Figure 4). Therefore, compared to DSBs at interstitial sites, which usually result in small deletions [65]–[67], DSBs near telomeres are much more likely to result in large deletions. In clone GFP-7F1, the inhibition of ATM kinase activity with KU55933 or knockdown of ATM expression with shRNA resulted in a small increase in large deletions at interstitial DSBs beyond that caused by I-*Sce*I endonuclease alone (6.9% and 4.0%, respectively). Combining KU55933 and shRNA knockdown resulted in a small additional increase in the frequency of large deletions beyond that seen with KU55933 alone (9.0%). A deficiency in ATM therefore has little effect on the frequency of large deletions at interstitial DSBs. Although ATM plays an important role in protecting DSBs from resection [7]–[11], [13], the frequency of large deletions at interstitial DSBs in ATM-deficient cells may be minimized by a corresponding deficiency in the processing of DSBs, because ATM is also involved in the activation of the MRE11/CtIP nuclease activity that is required for the processing DSBs [14], [16]–[18], [76]. The role of MRE11 in C-NHEJ and A-NHEJ is also partially independent of ATM [22], however, this is unlikely to affect the frequency of large deletions, because without ATM, any processing by MRE11 would result in limited resection due to a failure to activate BRCA1 [18], [77] and EXO1 [13], [21] in ATM-deficient cells.

A deficiency in ATM had a much greater effect on the frequency of large deletions at DSBs near telomeres. The treatment of clone GFP-6D1 with KU55933 increased the frequency of large deletions at DSBs near telomeres by an additional 17.9% beyond the already high frequency caused by I-*Sce*I endonuclease alone, so that 65.2% of the cells contain large deletions (Figure 4). Knockdown of ATM with shRNA in clone GFP-6D1 also increased the frequency of large deletions, although to a lesser extent than with KU55933, increasing the frequency of large deletions by an additional 7.9% beyond that seen with I-*Sce*I endonuclease alone. Combining KU55933 and shRNA knockdown resulted in a slight increase in the frequency of large deletions beyond that seen with KU55933 alone, so that 67.7% of the cells contain large deletions. The fact that KU55933 or knockdown of ATM does not prevent large deletions at DSBs near telomeres suggests that, unlike interstitial DSBs, the processing and resection of DSBs near telomeres is not dependent on ATM. In contrast, the increase in large deletions caused by a deficiency in ATM at telomeric DSBs suggests that ATM is involved in protecting DSBs near telomeres, as it is at interstitial DSBs.

### The effect of ATM deficiency on NHEJ at interstitial and telomeric DSBs

**Figure 5:**
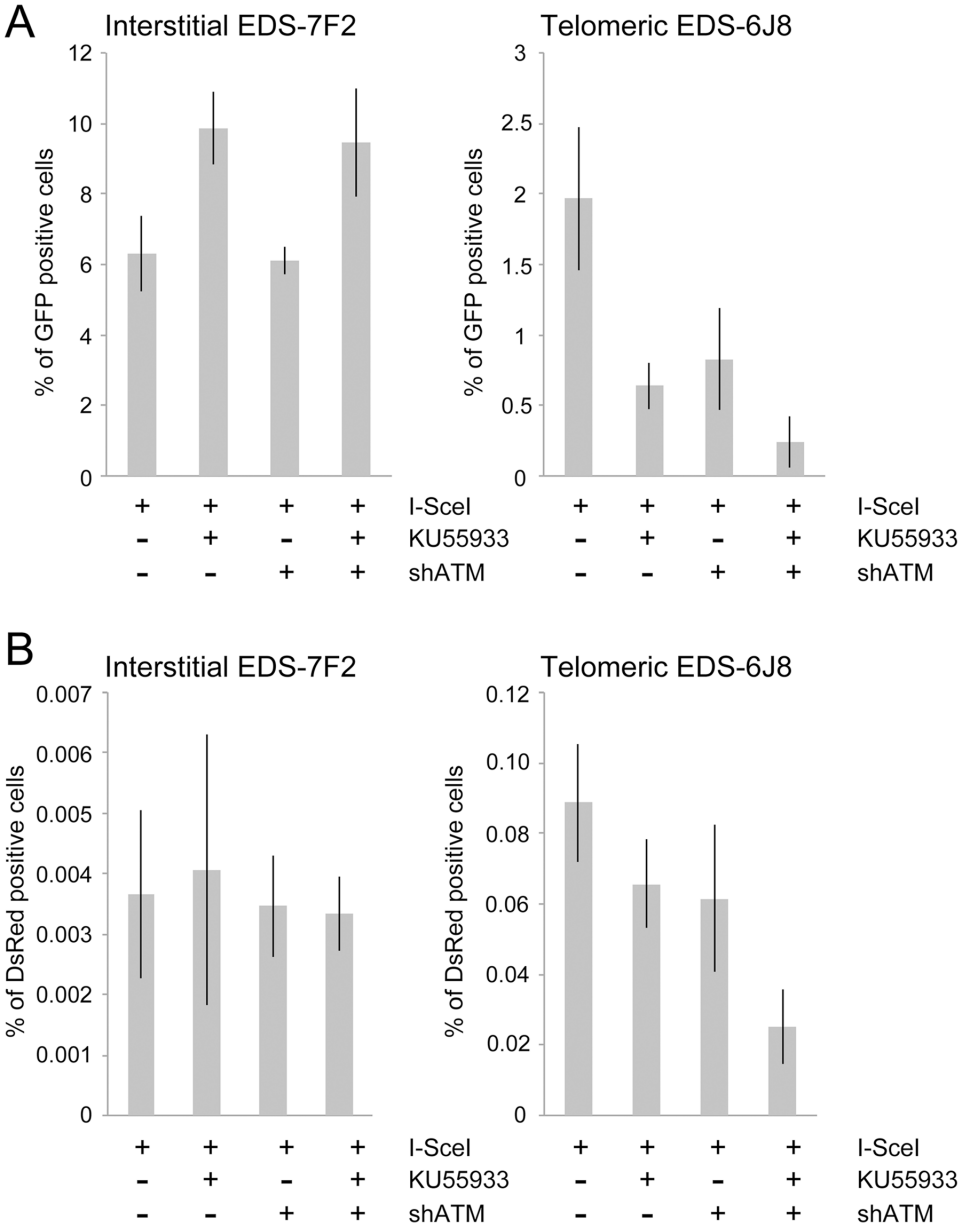
The effect of ATM deficiency on NHEJ and GCRs at interstitial and telomeric DSBs. (A) The frequency of GFP^+^ (NHEJ) and (B) the frequency of DsRed^+^ (GCRs) cells was determined in clones EDS-7F2 and EDS-6J8 following infection with the pQCXIH-ISceI retrovirus vector and selection with hygromycin for 14 days. All samples were analyzed in triplicate. Error bars represent standard deviation of three separate experiments.

The mechanism of repair of DSBs near telomeres was also investigated by comparing the effect of KU55933 and/or knockdown of ATM expression on the frequency of NHEJ (GFP^+^ cells) in clones EDS-7F2 and EDS-6J8 that contain the pEJ5-GFP plasmid integrated at interstitial or telomeric sites, respectively. Our results demonstrated that treatment with KU55933 caused a **57%** increase in the frequency of NHEJ in cell clone EDS-7F2, while the knockdown of ATM by shRNA caused no change in the frequency of NHEJ (Figure 5A).

Previous studies have found variable effects of KU55933 on NHEJ using similar assays. One study found no effect of KU55933 on NHEJ [23], one study found a decrease in NHEJ [22], and one study found an increase in NHEJ [78]. The latter study proposed that the increase in joining the distal ends of two I-*Sce*I-induced DSBs in ATM-deficient cells, as detected by this NHEJ assay, was a result of the loss of tethering of proximal ends of I-*Sce*I-induced DSBs. However, this conclusion is not consistent with our results showing that knockdown of ATM by shRNA had no effect on NHEJ at interstitial DSBs. Although we cannot rule out that the knockdown of ATM was insufficient to affect NHEJ, other studies have shown that kinase dead ATM can have very different effects from a deficiency in ATM. One study reported that inhibition of ATM kinase activity by KU55933 prevented HRR, while ATM deficient cells showed no change in HRR [79]. Mouse ES cells expressing kinase-deficient ATM were also found to have more chromosome instability than ATM knockdown cells, and the expression of kinase-deficient ATM results in embryonic lethality, while ATM knockout mice are viable [80], [81]. Therefore, because the kinase activity of ATM is dispensable for recruitment of ATM to damaged sites [80], [82], it was proposed that the displacement of ATM or NHEJ proteins from damaged sites requires ATM kinase activity, and that without this kinase activity the chromatin-associated ATM physically blocks the resection that is required for HRR [83]. Importantly, the inhibition of HRR can promote NHEJ [14], which could explain the increase in NHEJ in cells treated with KU55933 in our assay.

The effect of inhibition of ATM on NHEJ is very different at telomeric DSBs than it is at interstitial DSBs. Unlike clone EDS-7F2, KU55933 and/or knockdown of ATM expression in clone EDS-6J8 caused a large decrease in the already low frequency of I-*Sce*I-induced NHEJ (Figure 5A). The frequency of NHEJ was further decreased by combining KU55933 and knockdown of ATM, which together resulted in a nearly a 10-fold reduction in NHEJ. Importantly, the decrease in NHEJ near telomeres in ATM-deficient cells corresponded to the increase in large deletions (see Figure 4), consistent with a failure to protect DSBs near telomeres in ATM-deficient cells. Unprotected DSBs would result in increased resection, which would reduce the frequency of NHEJ in our assay, both because of degradation of the GFP gene, and because single stranded overhangs are poor substrates for C-NHEJ.

### The effect of ATM deficiency on GCRs at interstitial and telomeric DSBs

We next investigated the role of ATM in the formation of GCRs by analyzing the frequency of DsRed^+^ cells using the same EDS-7F2 and EDS-6J8 clones that were used for analysis of NHEJ. In clone EDS-7F2, the inhibition of ATM by KU55933 and/or shRNA-mediated knockdown of ATM caused no apparent change in I-*Sce*I-induced GCRs (DsRed^+^ cells, Figure 5B). These results suggest that ATM is not required for GCR formation at interstitial DSBs, although small changes in the frequency of GCRs may not be detected due to the very low frequency of DsRed^+^ cells at interstitial DSBs. It is not immediately clear why ATM would not be required for GCRs, because CtIP, which is activated by ATM, is required for GCRs [26]. One possibility is that despite the requirement for ATM in the activation of CtIP [14], [16]–[18], a deficiency in ATM would also eliminate the requirement for MRE11/CtIP for processing of DSBs due to the lack of end protection, which also requires activation by ATM [7]–[11]. Therefore, ATM-deficient cells may have sufficient processing of DSBs to provide a substrate for A-NHEJ without resulting in the extensive resection leading to large deletions. This possibility is consistent with the fact that ATM is required for HRR, which requires extensive resection, but is not required for the limited processing required by A-NHEJ [18], [77].

In contrast to clone EDS-7F2, the inhibition of ATM by KU55933 and/or shRNA-mediated knockdown of ATM in clone EDS-6J8 with a telomeric pEJ5-GFP plasmid caused a large decrease in I-*Sce*I-induced GCRs (Figure 5B). Moreover, similar to NHEJ, the decrease in the frequency of GCRs was additive when KU55933 and ATM knockdown were combined. Therefore, the decrease in GCRs near telomeres in ATM deficient cells, as is detected by this assay (those with relatively small deletions), is most likely a result of the increased frequency of large deletions due to excessive resection at the DSB. It is important to point out that these results do not mean that the inhibition of ATM prevents GCRs at DSBs near telomeres, because this assay only detects GCRs that occur with minimal degradation at the DSB, and large deletions at DSBs near telomeres commonly result in GCRs [68], [71].

### The effect of ATM deficiency on small deletions at interstitial and telomeric DSBs

**Figure 6:**
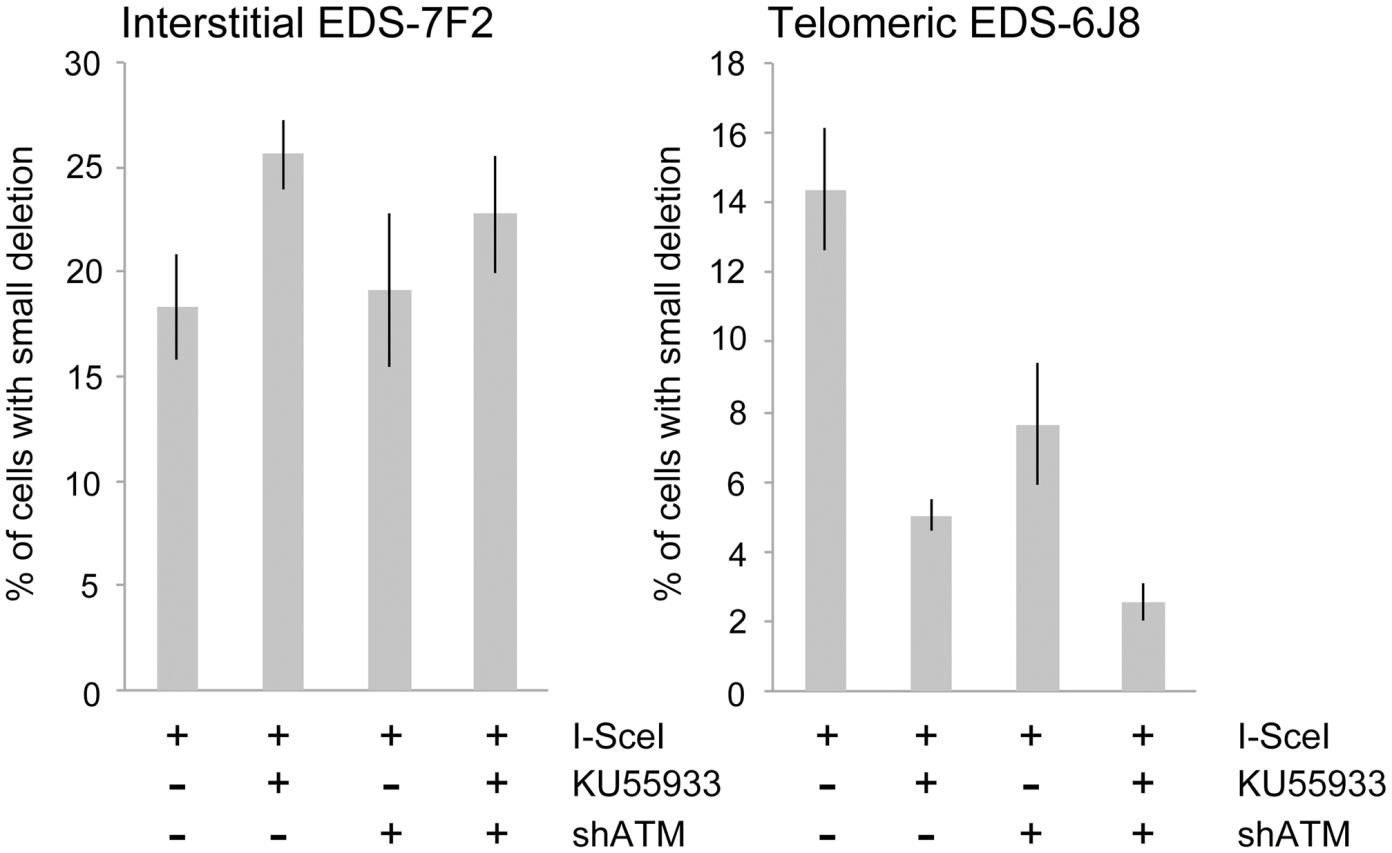
The effect of ATM deficiency on small deletions at interstitial and telomeric DSBs. The frequency of small deletions in genomic DNA from clones EDS-7F2 and EDS-6J8 that were infected with pQCXIH-ISceI and selected with hygromycin for 14 days was determined by first performing PCR using oligonucleotide primers spanning one of the I-*Sce*I sites in the pEJ5-GFP plasmid (see Figure 1). The fraction of cells in the population that contain small deletions at the I-*Sce*I site was then determined from the fraction of the PCR product that was not digested with I-*Sce*I (see Materials and Methods). All samples were analyzed in triplicate. Error bars represent standard deviation of three separate experiments.

We next determined the effects of ATM deficiency on the frequency of small deletions at one of the I-*Sce*I sites in the pEJ5-GFP plasmid following expression of I-*Sce*I endonuclease in clones EDS-7F2 and EDS-6J8 (Figure 6). Similar to the NHEJ assay, these small deletions also involve NHEJ, but unlike the NHEJ assay, the assay for small deletions only detects NHEJ events in which the I-*Sce*I site is lost. The actual number of DSBs at an I-*Sce*I site is much greater, since I-*Sce*I sites are commonly restored by NHEJ [64], [84]. As we previously reported [68], the frequency of small deletions at interstitial and telomeric DSBs is very similar (Figure 6). As a result, small deletions outnumber large deletions at interstitial DSBs, while they equal less than half the number of large deletions at telomeric DSBs (compare Figure 4 and Figure 6) [68]. Similar to NHEJ, the inhibition of ATM kinase activity with KU55933 in clone EDS-7F2 with an interstitial pEJ5-GFP plasmid caused a significant increase in the frequency of small deletions, while the knockdown of ATM expression by shRNA had no effect (Figure 6). Unlike clone EDS-7F2, a deficiency in ATM in clone EDS-6J8 with a telomeric pEJ5-GFP plasmid dramatically reduced the frequency of small deletions at the I-*Sce*I site, both with KU55933 and with knockdown by shRNA (Figure 6). The combined treatment with KU55933 and shRNA knockdown had an even larger effect, decreasing the percentage of cells with small deletions to only 2.6%. The effect of ATM deficiency on small deletions therefore mimics the effect of ATM deficiency on the NHEJ assay (Figure 5A), suggesting that small deletions at a single I-*Sce*I site and NHEJ between distal ends of two different I-*Sce*I sites occur through the same pathway. As with NHEJ, the decrease in small deletions corresponds to an increase in large deletions, strongly suggesting that small deletions near telomeres become large deletions in ATM-deficient cells. These results therefore add additional support for our conclusion that DSBs near telomeres require ATM for protection, but that the extensive processing and resection at DSBs near telomeres is not dependent on ATM.

## Discussion

The data presented here confirm and extend our earlier results that telomeric regions are highly sensitive to DSBs. In addition to the decrease in NHEJ and the increase in large deletions we have previously reported [56], [68], we now show that DSBs near telomeres have a much greater likelihood of generating GCRs. The frequency of GCRs is 20–50 fold higher at DSBs near telomeres compared to DSBs at interstitial sites (Figure 2). The effect of ATM deficiency on the types of events resulting from I-*Sce*I-induced DSBs also demonstrates important differences in the mechanism of DSB repair at interstitial and telomeric sites. Treatment with KU55933 or knockdown of ATM expression by shRNA caused a much larger increase in the frequency of large deletions at telomeric DSBs than at interstitial DSBs. This increase in large deletions would explain the corresponding decrease in small deletions and NHEJ at telomeric DSBs, both indirectly because of increased degradation, and directly because resected DNA is a poor substrate for C-NHEJ [11], [51], [60], [61].

**Figure 7:**
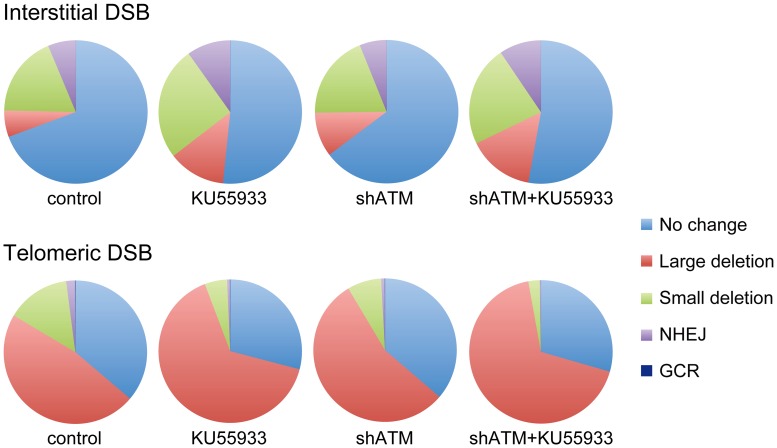
The relative proportion of different types of rearrangements resulting from interstitial and telomeric DSBs. The percentage of NHEJ, small deletions, GCRs and large deletions is shown for clones EDS-7F2 (interstitial pEJ5-GFP and interstitial pDsRed-ISceI) and EDS-6J8 (telomeric pEJ5-GFP and interstitial pDsRed-ISceI) following expression of I-*Sce*I for 14 days. Untreated control cultures are compared with cultures treated with KU55933, shRNA-mediated knockdown of ATM, or combined KU55933 and shRNA-mediated knockdown of ATM. The category of large deletions also includes chromosome healing and inversions; however, these events are very rare. The percentage of GCRs as measured by DsRed^+^ cells (those with small deletions) is too low to be visible in this figure.

Although most cells in the population show no rearrangements at the I-*Sce*I site in clones with interstitial DSBs (Figure 7), this does not mean that DSBs were not generated at the I-*Sce*I site in these cells, because the I-*Sce*I site is commonly restored during DSB repair [84]–[86]. Therefore, the predominance of large deletions in clones with telomeric I-*Sce*I sites (Figure 7) strongly suggests that I-*Sce*I sites that are restored by NHEJ at interstitial DSBs are much more likely to become large deletions at telomeric DSBs. This conclusion is consistent with our model in which DSBs near telomeres are prone to excessive resection that inhibits C-NHEJ [44], [56]. This model proposes that excessive resection at DSBs near telomeres results in GCRs involving A-NHEJ, because A-NHEJ requires single-stranded overhangs [18], [22]–[27]. A role for A-NHEJ at DSBs near telomeres is supported by the fact that A-NHEJ is associated with large deletions [23], [31] and GCRs [26], [30]–[33]. Excessive resection at telomeric DSBs would also explain why HRR is not deficient near telomeres [56], because HRR also requires extensive single-stranded 3′ overhangs [2].

The combined effect of ATM deficiency in our assays does not support the hypothesis that the sensitivity of telomeric regions to DSBs is due solely to the inhibition of ATM by TRF2, which would prevent the ATM-mediated changes that are required for repair of DSBs in heterochromatin [14], [87]. The fact that the inhibition of ATM increases the frequency of large deletions at telomeric DSBs demonstrates that ATM is functional near telomeres and would still be involved in the protection of DSBs, similar to its role at interstitial DSBs [7]–[11], although we cannot rule out the possibility that a partial inhibition of ATM near telomeres contributes to the sensitivity of telomeric regions to DSBs by decreasing end protection. Moreover, a localized deficiency in ATM would not in itself result in the high frequency of large deletions near telomeres, because the inhibition of ATM prevents resection of unprotected DSBs in G1 [11] and in heterochromatin in G2 [14]. Regardless, the observation that the inhibition of ATM did not prevent the formation of large deletions is consistent with our model in which large deletions at DSBs near telomeres occur by a mechanism that is different from the ATM-dependent processing and resection at interstitial DSBs. As an alternative, we have proposed that DSBs near telomeres are mistakenly processed as though they are telomeres, which involves Apollo and/or MRE11 and is mediated by TRF2, and is independent of ATM [44], [56]. This model is consistent with the observation that tethering TRF2 near interstitial DSBs inhibits their repair [63]. Unlike the processing of telomeres, the inability of POT1 to bind to the non-telomeric single-stranded 3′ overhangs at subtelomeric DSBs would result in extensive resection and GCRs involving A-NHEJ, as is the case with telomeres in POT1-deficient cells [53], [54], [88].

A role for A-NHEJ in repair of telomeric DSBs is supported by the presence of microhomology at the recombination junctions involved in GCRs resulting from DSBs near telomeres (Figure 3), since A-NHEJ commonly utilizes microhomology during end joining [22], [23], [27], [29], [30]. Microhomology has also been observed at recombination junctions involved in chromosome fusions resulting from DSBs near telomeres [68], [89] or telomere shortening [90]. However, one study found that although significant microhomology was found at chromosome fusions in fibroblasts in crisis and in the invasive ductal carcinoma stage of breast cancer, no significant microhomology was found in the ductal carcinoma in situ stage of breast cancer, suggesting that chromosome fusions commonly occur by C-NHEJ in early stages of breast cancer [91]. It is important to note, however, that the PCR-based assay used for analysis of chromosome fusion in this study does not detect the chromosome fusions described in our studies, which commonly involve large deletions that would result in the loss of the regions near telomeres required for PCR analysis.

Although a deficiency in ATM reduced the high frequency of DsRed^+^ cells that result from DSBs near telomeres in our assay system, it is important to point out that this does not mean ATM is required for A-NHEJ, which is involved in the formation of most GCRs [26], [30]–[33]. As mentioned earlier, this assay does not detect GCRs that involve large deletions, because GCRs with large deletions would not activate the DsRed gene. A deficiency in ATM causes a substantial increase in the frequency of large deletions at DSBs near telomeres (Figure 4), which commonly result in GCRs [68], [71]. Therefore, a deficiency in ATM would not result in an overall decrease in GCRs at DSBs near telomeres, only a decrease in GCRs with little or no degradation at the recombination junction.

The use of I-*Sce*I endonuclease to generate DSBs involves certain caveats and limitations that should be considered when interpreting the results. First, the direct ligation of I-*Sce*I-induced DSBs, which regenerates the I-*Sce*I site, is a common event [84]–[86] that is not detected by our assay systems. As a result, each I-*Sce*I site can be cut multiple times, which can alter the consequences of DSBs [85] and amplify the frequency of rearrangements at the I-*Sce*I site. Second, due to differences in accessibility of the I-*Sce*I endonuclease, I-*Sce*I-induced DSBs may be more likely to be generated at specific times in the cell cycle. Because repair of DSBs can differ during the cell cycle [92], this may affect the types of rearrangements that would occur. Lastly, the integration of plasmid DNA containing active promoters can affect chromatin conformation at the integration site, which could affect repair of DSBs, since chromatin conformation can influence DSB repair [14], [39], [41]. As a result, the use of integrated plasmids containing selectable marker genes cannot address differences in repair due to naturally occurring differences in chromatin conformation or transcription. Additional studies using DSBs generated by nonenzymatic means will therefore be required to confirm our results. One potential artifact that can be ruled out in our studies is that the difference in the type of events observed at interstitial and telomeric sites is due to a difference in the frequency of DSBs generated by I-*Sce*I endonuclease. Although subtelomeric regions are typically composed of heterochromatin [59], [72], [73], which could inhibit I-*Sce*I-induced DSB formation near telomeres, this difference in chromatin conformation has been minimized in our cell clones by prior selection for expression of the puro or GFP genes. In addition, although the lower frequency of NHEJ near telomeres could be explained by fewer I-*Sce*I-induced DSBs near telomeres, the frequency of GCRs and large deletions is increased near telomeres, and the frequency of small deletions and HRR is similar at telomeric and interstitial DSBs [56]. Moreover, a fundamental difference in repair of DSBs near telomeres is shown by the large size of the deletions at telomeric DSBs [68], [71], and by the different effects that ATM deficiency has on DSB repair at interstitial and telomeric sites.

Despite the caveats and limitations of assays based on I-*Sce*I-induced DSBs, the deficiency in repair of I-*Sce*I-induced DSBs near telomeres in the EJ-30 human tumor cell line appears to be representative of the response of mammalian cells to telomeric DSBs. Similar rearrangements were also observed in response to I-*Sce*I-induced DSBs near telomeres in mouse ES cells [89]. Moreover, the types of rearrangements observed as a result of I-*Sce*I-induced DSBs near telomeres are very similar to the types of rearrangements resulting from spontaneous telomere loss in the EJ-30 human tumor cell line [69], [70], [93]. The observation that a high rate of spontaneous telomere loss is common among human tumor cell lines [94] previously led us to propose that the sensitivity of telomeric regions to DSBs plays an important role in chromosome instability in human cancer [46]. Consistent with this proposal, telomere dysfunction resulting from oncogene-induced replication stress was subsequently found to cause senescence in normal cells, leading to the proposal that oncogene-induced telomere dysfunction can also serve as a protection against cancer in cells with intact cell cycle checkpoints [64]. Because most dysfunctional telomeres in the senescent cells in this study retained telomeric repeat sequences, the investigators concluded that oncogene-induced senescence results from persistent DSBs that occur near telomeres during replication stress. The deficiency in DSB repair near telomeres is also important in senescence resulting from ionizing radiation, as shown by the fact that persistent DSBs resulting from ionizing radiation co-localize with telomeres and correlate with radiation-induced cell senescence, both in cultured human primary fibroblasts and *in vivo* in mice [62], [63]. Although the precise location of the DSBs near telomeres was not determined in these studies, as we have previously pointed out [44], the frequency of the persistent DSBs suggests a target size that included subtelomeric DNA, consistent with our earlier studies demonstrating that the region that is sensitive to DSBs extends at least 100 kb from the telomere [71]. A similar sensitivity to DSBs near telomeres in human germ line cells would also explain the high degree of variability in subtelomeric regions in humans, which have been attributed to a high frequency of translocations [95]. In addition, the sensitivity of telomeric regions to DSBs could explain the prevalence of human genetic diseases resulting from terminal deletions and inversions at the ends of chromosomes that are associated with translocations [96]–[100]. Further studies in the mechanism of sensitivity of telomeric regions to DSBs should therefore provide valuable insights into the mechanisms of human disease.

## Materials and Methods

### Plasmids

The pEJ5-GFP plasmid (Figure 1A) has previously been used to monitor the frequency of NHEJ at telomeric and interstitial DSBs [27], [56]. The pGFP-ISceI plasmid (Figure 1A) has previously been used to monitor the frequency of large deletions at telomeric and interstitial DSBs [56]. The pGFP-ISceI plasmid was generated from the pEJ5-GFP plasmid by deletion of the puromycin-resistance (puro) gene following NHEJ between the two I-*Sce*I sites. The pDsRed-ISceI plasmid (Figure 1B) was created by inserting an 18 bp recognition site for I-*Sce*I endonuclease between the *Bgl*II and *Eco*RI restriction sites at the 5′ end of the promoterless DsRed gene in the pDsRed-Express-1 plasmid (Clontech).

### Cell lines

All of the cell lines used in this study were derived from clone B3 of the EJ-30 human bladder cell carcinoma cell line. EJ-30 is a subclone of the EJ human colon cancer cell line, which is also called MGH-U1 [101]. The cells were grown in MEM alpha media (UCSF Cell Culture Facility) supplemented with 5% fetal calf serum (Invitrogen-Gibco), 5% newborn calf serum with iron (Invitrogen-Gibco), 1 mM *l*-glutamine (Invitrogen-Gibco), and were propagated at 37°C in humidified incubators.

The GFP-7F1 and GFP-6D1 clones containing the pGFP-ISceI plasmid integrated at interstitial and telomeric sites, respectively, were previously used to investigate the frequency of large deletions [56]. The EJ5-7F and EJ5-6J clones containing the pEJ5-GFP plasmid integrated at interstitial and telomeric sites, respectively, were previously used to investigate the frequency of NHEJ [56]. Clones EJ5-7F and EJ5-6J were transfected with the pDsRed-ISceI plasmid linearized with *Apa*LI, and colonies containing the stably integrated pDsRed-ISceI were selected with G418. The number of integrated copies of the pDsRed-ISceI plasmid was then determined by Southern blot analysis using a variety of restriction enzymes (see below). We identified four EJ5-7F clones that contain a single copy of the pDsRed-ISceI plasmid (EDS-7F2, EDS-7F4, EDS-7F5, and EDS-7F6), six EJ5-6J clones that contain a single copy of the pDsRed-ISceI plasmid (EDS-6J8, EDS-6J29, EDS-6J31, EDS-6J35, EDS-6J41, EDS-6J49), and two EJ5-6J clones containing three copies of the plasmid (EDS-6J7, EDS-6J10).

### Generation of I-*Sce*I-induced DSBs

Packaging of the pQCXIH and pQCXIH-ISceI retroviral vectors and infection of cell cultures was performed as previously described [68]. The selection for cells infected with pQCXIH-ISceI was achieved by growth in medium containing 50 µg/ml hygromycin (Sigma) for 14 days with medium changes every 2 days to allow for expression of I-*Sce*I endonuclease and the generation of DSBs. After 12 days, the cells were trypsinized and replated. After an additional 2 days, the cells were trypsinized again, pooled, and either analyzed for the frequency of GFP-positive (GFP^+^) and DsRed-positive (DsRed^+^) cells, or replated for preparation of genomic DNA.

### Inhibition of ATM by KU55933 and shRNA knockdown

KU55933 is an effective inhibitor of ATM kinase activity [102]. Treatment of cells with 10 µM KU55933 began the day after infection with pQCXIH or pQCXIH-ISceI and continued during the 14-day period prior to cell analysis using our assays. The shRNAs for knockdown of gene expression were introduced into cells using the pSiren RetroQ-Blasticidin retrovirus vector (kindly provided by Denise Chan, UCSF). The pSiren RetroQ-Blasticidin retrovirus vector was generated from the pSiren RetroQ retrovirus vector (Clontech) by replacing the puro gene with the blasticidin-resistance gene. The packaging the retrovirus vector was performed as previously described [68]. The shRNA sequence for knockdown of ATM was 5′-GCAACATACTACTCAAAGA-3′, which has previously been shown to effectively knockdown expression of ATM [103]. The efficiency of knockdown of ATM gene expression was determined by quantitative real-time PCR (see below). The efficiency of knockdown was 88.5% for clone EDS-7F2, 70.7% for clone EDS-6J8, 80.5% for clone GFP-7F1, and 75.9% for clone GFP-6D1.

### Quantitative real-time PCR

RNA isolation for analysis of gene expression was performed using an RNeasy kit (Qiagen) following the manufacturer's instructions. cDNA was generated from 1.5 µg of total RNA, using M-MLV-RT (Invitrogen) following the manufacturer's instructions. Quantitative real-time PCR was performed on cDNA samples using a StepOnePlus Real-Time PCR machine (Applied Biosystems). PCR was performed using 2.5 µl of the cDNA sample, 0.2 µl of 10 µM forward primer, 0.2 µl of 10 µM reverse primer, and 5 µl of Power Sybr Green PCR Master Mix (Applied Biosystems) following the manufacturer's instructions. A mixture of cDNA from cell clones EDS-6J8, EDS-7F2, GFP-6D1 and GFP-7F1 that was undiluted, diluted 4X, 16X, and 64X, was used as a standard. The level of expression of the housekeeping gene GAPDH was also analyzed in each sample to control for the efficiency of PCR in each sample. The knockdown efficiency of ATM was calculated by comparing the expression level of the ATM and GAPDH genes in cell cultures with and without the shRNA for ATM. The expression level of the ATM and GAPDH genes were calculated by absolute quantification relative to the standard curve using the Standard Curve Method with the SDS software provided by the manufacturer (Applied Biosystems).

The primers used for analysis of ATM expression by quantitative real-time PCR were ATM-F, 5′-TCCAGGGGAAGATGATGAAGA-3′, and ATM-R, 5′-TCTACAATGAGCTGCGTGTGG-3′. The primers used for analysis of GAPDH expression used as an endogenous control were GAPDH-F, 5′-GTTGCCATCAATGACCCCTT-3′, and GAPDH-R, 5′-ACTCCACGACGTACTCAGCG-3′.

### Flow cytometry of GFP^+^ and DsRed^+^ cells

The analysis of the frequency of GFP^+^ and DsRed positive (DsRed^+^) cells was performed using an Accuri C6 Flow Cytometer (BD Biosciences). The cells were trypsinized, an equal volume of growth medium was added, and the cells were counted and pelleted. To prevent aggregation, the cells were then resuspended in 10 ml of ice-cold Dulbecco's PBS (w/o Ca or Mg) containing 100 µg/ml Proteinase K (Sigma) by vigorous pipeting with a fine bore plastic pipet. The cells were then incubated 10 min on ice, pipeting twice more during the incubation. This treatment with Proteinase K is necessary with EJ-30 to keep the cells from aggregating. Following the incubation 2 ml of Dulbecco's PBS (w/o Ca or Mg) containing 1% BSA (Sigma) was added to block further digestion with Proteinase K. The cells were then pelleted and resuspended in Dulbecco's PBS (w/o Ca or Mg) at approximately 1×10^6^ cells/ml for analysis by flow cytometry. Approximately 1×10^6^ cells were counted for each sample. All samples were analyzed in triplicate. Error bars represent standard deviation of experiments that were conducted three times.

### PCR analysis and sequencing of recombination junctions

DNA sequence analysis of recombination junctions involved in activation of the DsRed gene was accomplished by first isolating pooled populations of DsRed^+^ cells by flow sorting (in conjunction with the UCSF Cell Analysis Core Facility) from clones EDS-6J7 and EDS-6J8 that were selected for 14 days with hygromycin following infection with the pQCXIH-ISceI retrovirus. The pooled populations of DsRed^+^ cells were then plated as single cells and allowed to grow into colonies, after which individual colonies were selected at random. The genomic DNA from the subclones generated from the various colonies was then isolated and amplified by PCR using the EJ5-1 primer, 5′-ATGGTAATCGTGCGAGAGGG-3′, located at the end of the promoter in the EJ5-GFP plasmid, and the DSR-1 primer, 5′-TGAAGCGCATGAACTCCTTG-3′, located at the 5′ end of the DsRed gene (see Figure 1B). The conditions for PCR involved 94°C for 2 min, then 40 cycles of 94°C for 30 sec, 62°C for 30 sec, and 72°C for 45 sec. DNA sequence analysis was then performed directly on the PCR products using the EJ5-1 primer (MCLAB).

### Analysis of large deletions

The frequency of GFP^+^ cells was determined using a Cellometer Vision (Nexelcom). The cells were first trypsinized and 20 µl of growth medium containing approximately 1×10^4^ cells was aliquoted into a counting chamber slide (Nexelcom). Two counting chambers were used for each sample, with each chamber being counted two times. All samples were analyzed in triplicate. Error bars represent standard deviation of experiments that were conducted three times. The results with the Cellometer Vision were verified by visual analysis of the cells being counted, and were very similar to results obtained using flow cytometry (data not shown).

### Analysis of small deletions

The presence of small deletions at a single I-*Sce*I-induced DSB were analyzed by first generating PCR products spanning an I-*Sce*I site in the integrated pEJ5-GFP plasmid, and then digesting the PCR products with I-*Sce*I endonuclease. PCR was performed on genomic DNA isolated from the pooled hygromycin-resistant cell cultures 14 days after infection with the pQCXIH-ISceI retroviral vector. PCR was performed using Taq 2X Master Mix (New England Biolabs) and primers GFP-1 (5′-GCGGGGTTCGGCTTCTGG-3′) and GFP-3 (5′-CGCTTCCATTGCTCAGCGG-3′) (see Figure 1A). PCR involved 94°C for 2 minutes, then 40 cycles of 94°C for 30 seconds, 62°C for 30 seconds, and 72°C for 30 seconds. 25 µl of the PCR product was then digested with 20 units of I-*Sce*I endonuclease at 37°C overnight, and the products were run on 4% agarose gels. After staining with ethidium bromide, digital images were analyzed using Image J software (http://download.cnet.com/ImageJ/3000-2192_4-37303.html?tag=vtredir) to calculate the intensity of the bands. The fraction of cells containing small deletions (SD) at the I-*Sce*I site was determined by dividing the intensity of the uncut band (UC) by the combined intensity of the cut (C) and uncut bands. The values for small deletions were then corrected for the fraction of cells that had large deletions or NHEJ, because these cells would not produce a PCR product, and would therefore cause an overestimation of the fraction of cells containing small deletions. The fraction of cells with small deletions therefore involves multiplying the fraction of uncut PCR product by 1 minus the fraction of cells with large deletions (LD), as determined in our large deletion assay, and by 1 minus the fraction of cells with NHEJ, as determined by our NHEJ assay. The final equation for the fraction of cells with small deletions is therefore: SD = UC/UC+C×(1−LD)(1−NHEJ). Although inversions of the fragment between the two I-*Sce*I sites would also prevent small deletions, this was not corrected for because the frequency of these events is too low to significantly affect our results [31]. The validity of this correction was previously demonstrated by the analysis of the frequency of small deletions in 100 individual subclones selected at random [68]. All samples were analyzed in triplicate. Error bars represent standard deviation of experiments that were conducted three times.

### Southern blot analysis—determining DsRed copy number

Southern blot analysis was performed on genomic DNA isolated from the individual G418-resistant subclones transfected with the pDsRed-ISceI plasmid to determine the number of integrated plasmid copies. Southern blot analysis was performed as previously described [104] using the pDsRed-ISceI plasmid as a probe. Copy number was determined by digesting genomic DNA with three separate restriction enzymes, *Bam*HI, *Bgl*II, and *Eco*RI, that cut once in the plasmid adjacent to the I-*Sce*I site. Clones containing a single copy of pDsRed-ISceI will show two plasmid-specific bands with each of the three restriction enzyme digests.
